# The Effect of Filtration on Physical and Chemical Properties of Osmo-Dehydrated Material

**DOI:** 10.3390/molecules25225412

**Published:** 2020-11-19

**Authors:** Klaudia Masztalerz, Adam Figiel, Anna Michalska-Ciechanowska, Aneta Wojdyło, Paulina Nowicka, Krzysztof Lech

**Affiliations:** 1 Institute of Agricultural Engineering, Wroclaw University of Environmental and Life Sciences, Chełmońskiego 37/41, 51-630 Wroclaw, Poland; klaudia.masztalerz@upwr.edu.pl (K.M.); krzysztof.lech@upwr.edu.pl (K.L.); 2 Department of Fruit, Vegetable and Nutraceutical Plant Technology, Wroclaw University of Environmental and Life Sciences, Chełmońskiego 37, 51-630 Wroclaw, Poland; anna.michalska@upwr.edu.pl (A.M.-C.); aneta.wojdylo@upwr.edu.pl (A.W.); paulina.nowicka@upwr.edu.pl (P.N.)

**Keywords:** filtration, bioactivity, antioxidant capacity, SEM, water loss, solid gain

## Abstract

Osmotic dehydration (OD) performed in concentrated fruit juices used as osmotic solution (OS) comes with some limitations resulting from the material cell structure and is not entirely recognized at the moment. Filtration of the juice could provide some insight into the phenomena occurring throughout the OD. Therefore, the main aim of the study was to recognize the mechanism of selective penetration during OD and evaluate the effect of filtration on physical and chemical properties of osmo-dehydrated material. For this purpose, OD of pumpkin in non-filtrated and filtrated (filters 0.2, 0.45, 0.8, 1.2, 3, 5 and 8 μm) concentrated chokeberry juice was carried out in the study. Moreover, scanning electron microscope (SEM) images were provided. Total phenolic content (TPC) and antioxidant capacity measured by Ferric Reducing Antioxidant Potential (FRAP) and Trolox Equivalent Antioxidant Capacity (TEAC ABTS) of OS and the material were determined. It was found that even though filtration of osmotic solution had a moderate influence on the mass transfer, it greatly affected the chemical composition of dehydrated material. The best option, considering both chemical and physical properties of the dehydrated material, is the use of non-filtrated solution. However, when shorter time of OD is considered, much better results are obtained for filtrated solutions.

## 1. Introduction

Osmotic dehydration (OD) is one of the drying methods usually applied as a pretreatment. The final moisture content of the osmotically dehydrated material is still high and thus, the material is prone to microbiological deterioration [1,2]. However, as a result of reduced moisture content, osmotic dehydration decreases the time and cost of drying as well as prolongs the shelf-life of dehydrated products [3]. Depending on the type of osmotic solution, in addition to dehydration, OD can also be used to create new products with improved quality and new properties [4]. Among many factors influencing osmotic dehydration are concentration and temperature of osmotic solution [5], duration of the process [6], type and geometry of the sample [7] and many others. Yet, as the process occurs between the osmotic solution and biological material, naturally, it comes with some limitations resulting from the material cell structure and size of the capillaries and other pathways involved in the process that can affect the OD [8].

Several mechanisms of mass transfer between hypertonic solution and porous cellular material take place during osmotic dehydration. The main driving force of the process is the difference between osmotic pressure inside the cell and surroundings known as turgor pressure. Due to the shrinking occurring in the course of OD, the plasmalemma is detached from the cell wall, which is called plasmolysis. Consequently, uncontrollable inflow of osmotic solution takes place.

Due to the limited size of the pathways involved in mass transfer, such as gas-filled capillaries, cell walls and intercellular and extracellular spaces, the size of the particles that can be transported into the sample in the course of osmotic dehydration can play an important role in the process [8]. It has been reported that solids influx is facilitated by low molecular weight solutions, unlike high molecular weight ones that tend to have higher viscosity and might get stuck in the outer part of the sample. Also, bigger particles are slower to move, which intensifies the tendency of these particles to agglomerate near the surface of the material [9]. Cichowska et al. [10] in the studies on osmotic dehydration of apples in polyols solutions stated that molecular weight of the osmotic solution is an important factor influencing osmotic dehydration. Also, the study revealed that high molecular weight particles can lower osmotic pressure and consequently hinder mass transfer during OD. The study on the OD of apple, banana and kiwi in glucose and sucrose solution showed that low molecular weight solute (glucose) contributed to higher water loss (WL) and solid gain (SG) than high molecular weight solute (sucrose) [11]. In the studies by Lech et al., it was observed that the pore size of the material might influence the mass transfer of the solution into the sample [12]. Lenart et al. stated that the addition of low molecular weight substances promoted water removal from the material [2]. Saurel et al. reported that when OD of apple was considered with an increase of solute molecular weight, the solid uptake decreased [13].

All of the above-mentioned reports highlighted that the size of the particles contained in the osmotic solution plays an important role in modelling of OD. When simple solutions (e.g., salt or sugar solutions) are used, it is easy to predict the chemical composition of the osmotic solution and possible effect on the dehydrated material. Fruit juices are not homogenous in their structure and as such, affect osmotic dehydration in a different way that is not entirely understood and examined at the moment. Yet, filtration of the osmotic solution, and as a result, removal of certain size particles, can lead to a better understanding of selective penetration during OD. Lech et al. studied the chemical properties of filtrated chokeberry juice used as osmotic solution [14]. The study showed that the size of particles in the osmotic solution can influence polyphenolic content and antioxidant capacity of the solution. The authors also suggested that further research should be carried out to find out whether the size of the particles in osmotic solutions prepared from concentrated juices can influence the polyphenolic content and antioxidant capacity of osmo-dehydrated material.

The effect of filtration on the distribution of solids inside the dehydrated material might be assessed using scanning electron microscope (SEM). SEM imaging has been previously used in the studies on OD of potatoes [15], pumpkin [16] and beetroots [17].

Butternut pumpkin has been used in this study due to its homogeneity and relatively long shelf-life, which makes it a perfect model material. Also, pumpkin has been studied before by Lech et al. [12].

The study aimed at recognizing how filtration of certain size particles influences the mass transfer during OD and how filtration of larger sized compounds affects the chemical composition, such as total polyphenolic content and antioxidant capacity of the sample during osmotic dehydration. Therefore, the main aim of the study was to examine the effect of different filtrates (filters 0.2, 0.45, 0.8, 1.2, 3, 5 and 8 μm) and non-filtrated solution prepared from concentrated chokeberry juice on the quality of dehydrated pumpkin *cv.* Butternut cylinders.

## 2. Results and Discussion

### 2.1. Physical and Chemical Properties of Osmotic Solutions Before the Osmotic Dehydration

Table 1 shows the physical and chemical parameters of filtrated and non-filtrated (NF) chokeberry juices used in the study. Density of osmotic solutions (OS) is similar in each case, which means that filtration of juices had no significant effect on the density of the solution.

Water activity of OS has been affected by filtration with significantly different NF osmotic solution and lower values obtained for the osmotic solutions with smaller particles (OS < 1.2 μm). Water activity ranged from 0.9424 ± 0.0008 in the case of NF osmotic solution to 0.9455 ± 0.0001 in the case of OS 0.2 μm. However, osmotic solution with the smallest particles (OS 0.2 μm) demonstrated the highest water activity (a_w_). Water activity affects the osmotic pressure and thereby the mass transfer during the process [2], and hence, it is an important factor influencing the overall course of OD.

As for viscosity, the osmotic solutions with smaller molecules showed a significant reduction of viscosity. Lower viscosity facilitates transport of the osmotic solution particles into the sample, while higher viscosity results in agglomeration of these particles and might drive them to stick to the outer part of the sample, therefore blocking the transfer of OS into the intercellular spaces [18,19].

In general, filtration of concentrated chokeberry juice resulted in lower amounts of total polyphenolic content (TPC) and lower values of antioxidant capacity measured by TEAC ABTS and FRAP (Table 1). It can be explained by the fact that high molecular weight compounds including tannins influence the total polyphenolic content [20], and therefore the highest TPC was reported for NF solution. It means that smaller particles remaining after separation contain lower contents of TPC measured by the Folin method. However, the lowest TPC was obtained in the case of OS 0.8 μm and OS 0.45 μm, whereas the solution with the smallest particles (OS < 0.2 μm) proved to contain the second highest TPC right after NF solution. Obtained results are inconsistent with studies on filtrated osmotic solution by Lech et al., where the smallest fraction of filtrates (OS 0.2 μm) contained one of the lowest amounts of TPC [14]. This discrepancy might be due to the heterogenous structure of concentrated chokeberry juice that as a biological material is likely to demonstrate slight differences in chemical composition as a result of many factors, i.e., environmental (harvest time, maturation, etc.) and processing conditions [21].

There are many studies showing the correlation between TPC and antioxidant capacity [22,23]. However, it is not always the case as substances other than phenolic compounds can increase antioxidant capacity of the sample while the TPC stays low [24]. Even though filtration reduced TPC in this study, it did not necessarily result in simultaneous reduction of antioxidant capacity. In several cases, TEAC ABTS and FRAP did not decrease accordingly, especially in the case of OS 0.8 μm when the FRAP value was significantly higher than in the case of non-filtrated solution (35.26 ± 0.87 mmol Trolox (Trx)·100 g^−1^ dm for OS 0.8 μm and 32.9 ± 0.81 mmol Trx·100 g^−1^ dm for NF OS). It should be noted that while OS 0.8 μm contained the lowest TPC, it also had the highest antioxidant capacity measured by FRAP and one of the highest measured by TEAC ABTS. It might be due to the presence of low molecular weight polyphenolic compounds and others that still present antioxidant capacity. Nevertheless, the antioxidant capacity measured by both methods decreased after filtration in comparison to the NF solution (with the above-mentioned exception of OS 0.8 μm in the case of FRAP). High initial ABTS of NF solution might be due to the presence of high molecular weight tannins that are much more potent than simple monomeric phenolics [20], but also, it can be a result of the concentration in which the antioxidants occur in the solution.

It should be noted that filtrated solutions were subjected to additional thermal treatment due to the evaporation of diluted solution right after the filtration. This might lead to degradation of thermolabile compounds and therefore reduce the TPC and antioxidant capacity of filtrated solutions (compared to initial raw material).

### 2.2. Osmotic Dehydration

**Figure 1:**
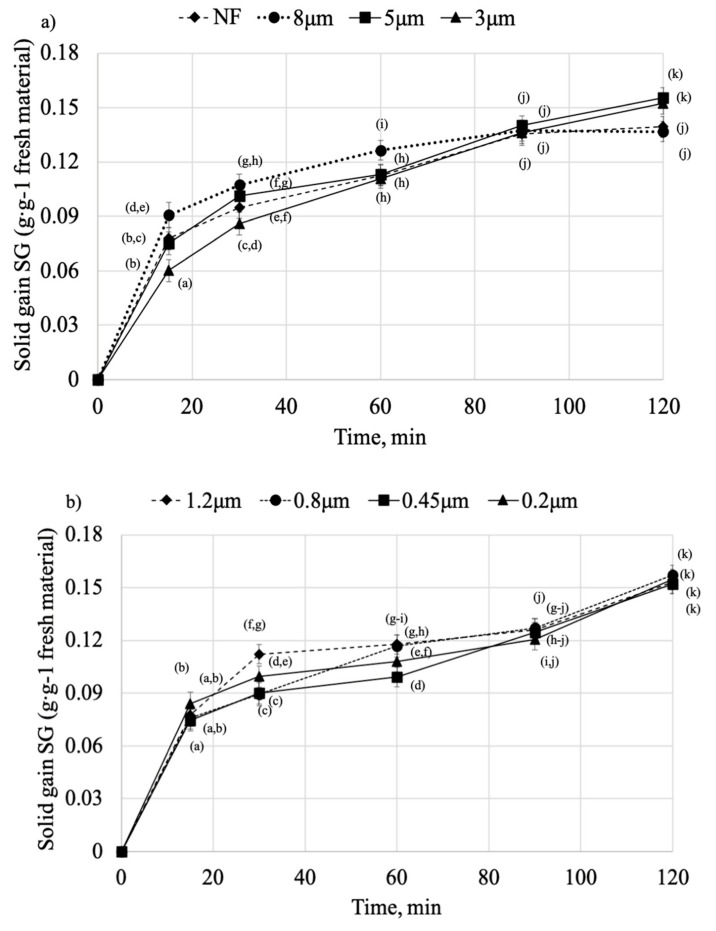
Solid gain (SG) of the pumpkin cylinders dehydrated in different osmotic solutions: (**a**) OS > 3 μm, (**b**) OS < 3 μm. Mean values followed by the same letter were not significantly different (*p* < 0.05) according to the HSD Tukey’s least significance difference test.

Solid gain (SG) of pumpkin cylinders dehydrated in filtrated and non-filtrated osmotic solutions is shown in Figure 1a,b. During the first 15 min of the process, the SG of every variant is very intensive, then the process rate diminishes, and with little increment, proceeds until the end of the process. This behavior is typical for OD, where at the beginning, the rate of SG is very high, and then decreases over time. It might be due to the decrease of osmotic pressure difference between solution and the material as a result of mass transfer and dilution of OS [6]. Similar behavior was noticed in the studies on pumpkin [18] and pomegranate seeds dehydrated in date juice [25]. Rapid solid gain during the first stage of dehydration (<30 min) results in high concentration of the solution on the surface of the sample that could hinder the countercurrent flow of water as well as impede further inflow of solids from osmotic solution. Formation of this layer on the outer part of the material has been previously reported, e.g., in the studies on potatoes [15].

At the beginning of the OD, a significant difference (*p* < 0.05) can be seen in SG of the samples dehydrated in non-filtrated (NF) and filtrated (OS > 3 μm) osmotic solutions. The sample treated with OS 8 μm obtained the highest SG after 15 min, and this tendency was maintained for the first 90 min of the process, after which the influx of particles slowed down. As for the sample dehydrated in OS 5 μm at the beginning, the process rate was moderate, then accelerated, at the end reaching one of the highest SG (SG = 0.1555 g·g^−1^ fresh material). Similar results were obtained for OS 3 μm, whereas non-filtrated (NF) and OS 8 μm samples showed the lowest values of SG after 120 min (SG = 0.1395 g g^−1^ fresh material and SG = 0.1369 g g^−1^ fresh material for NF and OS 8 μm, respectively).

When considering samples dehydrated in filtrates OS < 3 μm (Figure 1b), at the beginning, there is no significant difference between the process rate, and after 15 min, the samples treated by OS 1.2 μm exhibited higher SG while other variants demonstrated balanced or low SG throughout the process. However, at the end, all the samples dehydrated in filtrates OS < 3 μm reached almost the same value of SG (SG > 0.15 g g^−1^ fresh material). Previous work by Lech et al. [12] showed that low water activity of osmotic solutions resulted in a bigger difference of osmotic pressure between the sample and OS and therefore, led to higher SG. However, in this study, in the case of OS 0.2 μm, water activity was very high, yet similar SG was obtained. This might be explained by the lowest viscosity among all osmotic solutions that facilitated the mass transfer despite high water activity. Also, long time of thermal processing might result in loss of semi-permeability of the membrane and lead to higher SG after 90 min of the process [26].

Overall, filtration had a limited effect on SG. At the end, the highest SG was obtained for the sample treated by OS 0.8 μm (SG = 0.157 g·g^−1^ fresh material). Low molecular weight osmotic solutions are known to facilitate the OD as small particles penetrate into the sample easier than high molecular weight compounds [9]. On the other hand, the lowest SG was obtained for NF and OS 8 μm. It might be due to the high particle size of the juice that accumulated at the outer part of the sample, clogging the capillaries and hindering the mass transfer [27]. However, if 60 min OD is considered, then the sample treated by OS 8 μm showed the best results.

**Figure 2:**
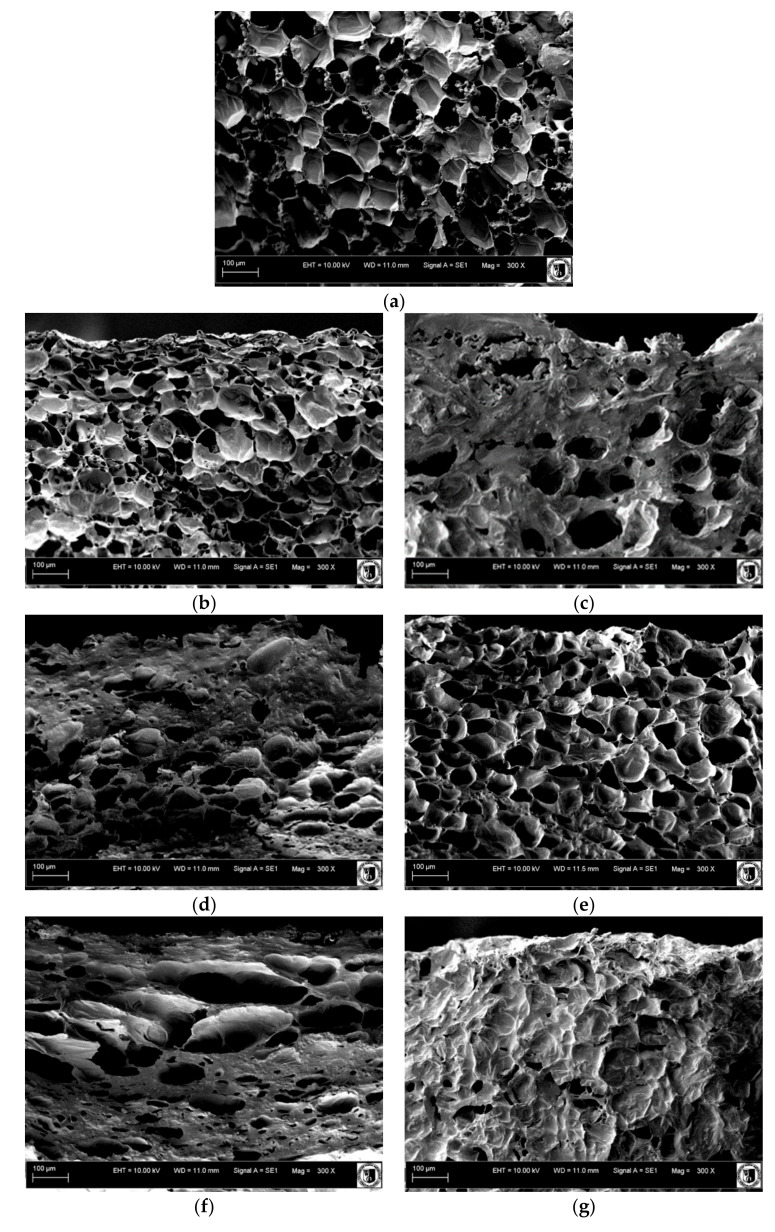
Scanning electron microscope (SEM) imaging of the pumpkin cylinders (**a**) before the OD, (**b**) after 15 min of OD in NF solution, (**c**) after 120 min of OD in NF solution, (**d**) after 15 min in OS 8 μm, (**e**) after 120 min in OS 8 μm, (**f**) after 15 min in OS 0.2 μm, and (**g**) after 120 min in OS 0.2 μm.

SEM images of the sample before and after OD shows that after 120 min of OD in OS 0.2 μm, the sample was evenly filled with solution, whereas when NF solution was applied, the openings in the pumpkin cylinders were still visible and an additional layer of solution on the surface of the material could be seen (Figure 2).

**Figure 3:**
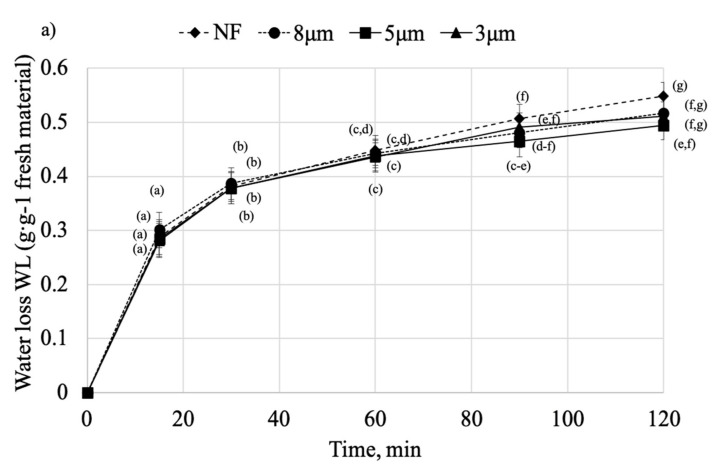

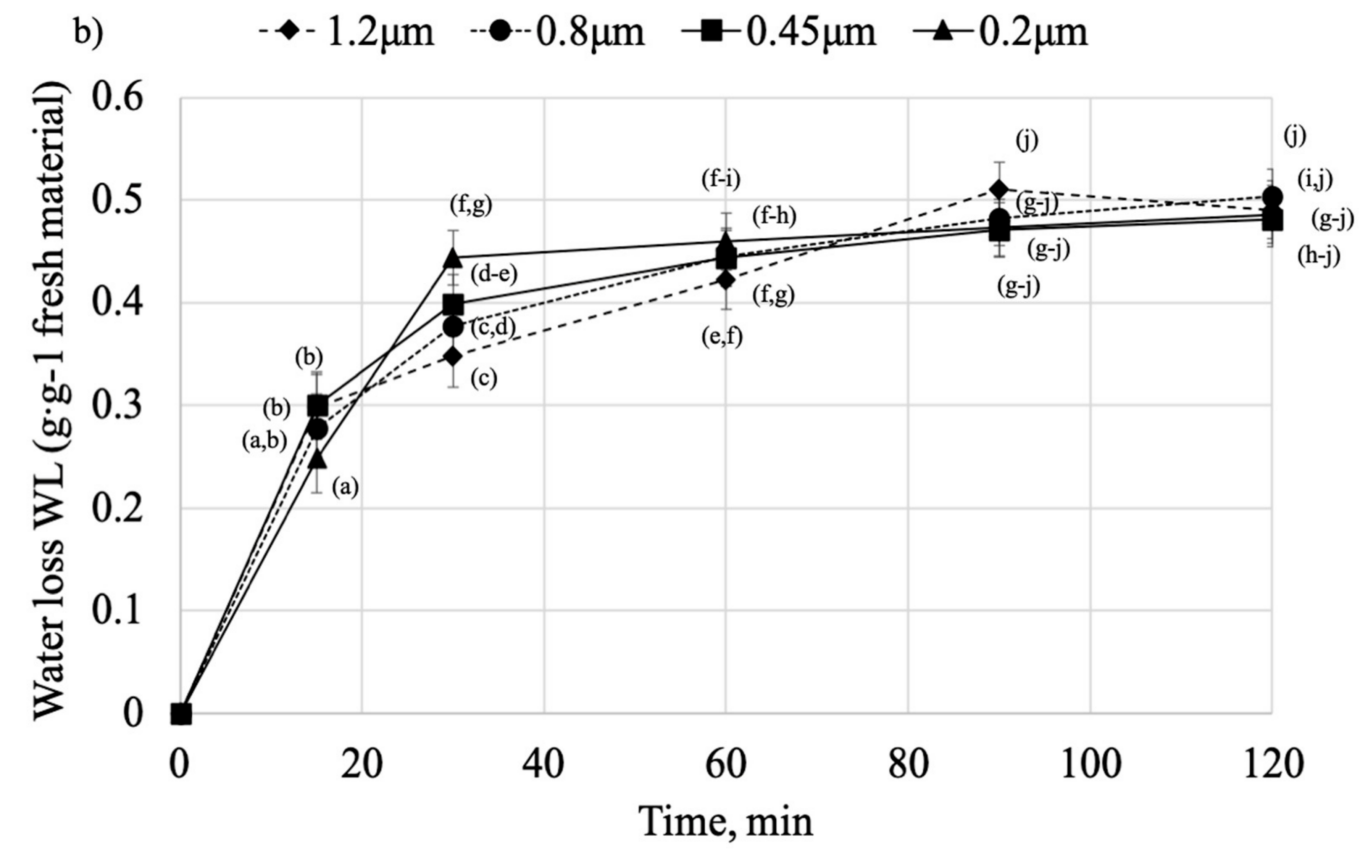
Water loss (WL) of the pumpkin cylinders dehydrated in different osmotic solutions: (**a**) OS > 3 μm, (**b**) OS < 3 μm. Mean values followed by the same letter were not significantly different (*p* < 0.05) according to the HSD Tukey’s least significance difference test.

The water loss (WL) of the samples dehydrated in different solutions is presented in Figure 3a,b. As can be seen, there is no significant difference in WL of the samples dehydrated in non-filtrated (NF) and OS > 3 μm during the first 60 min of the process (Figure 3a). Then, a significant increase (*p* < 0.05) of WL for NF samples can be seen after 90 min (WL = 0.548 g g^−1^ fresh material). As for OS 8 μm and OS 5 μm, lower WL at the end of the process might be due to the high SG which occurred at the same time, which could disrupt the mass transfer and reduce the WL. The highest WL reported for OS > 3 μm and NF solutions might be due to the high molecular weight of the particles inside these solutions that favors the WL over SG [28].

Meanwhile, when considering Figure 3b, there is a significant difference (*p* < 0.05) between solutions, with the highest WL increase during the first 30 min obtained when OS 0.2 μm was applied (WL_0.2 μm_ = 0.444 g g^−1^ fresh material compared to WL_1.2 μm_ = 0.348 g g^−1^ fresh material). It suggests that solutions with smaller particles strongly influence WL at the beginning of the process. It might be due to the higher osmotic pressure occurring when small molecular weight solutes are applied. Saurel et al. reported that OD is more intensive at the initial stage of the process when low molecular weight particles are involved [13]. Among other factors influencing the process might be high osmotic pressure and rapid WL at the beginning, that can result in shrinking of the sample and detachment of plasmodesmata that facilitates the influx of osmotic solution into the sample [8]. Also, lower resistance of small particles could promote the countercurrent flow of water from leaving the material. However, after 30 min, the WL of OS 0.2 μm declines and remains steady until the end of the process. Still, the growth of the WL for the OS 1.2 μm and 0.8 μm can be seen after 90 min, yet it is lower than for the samples dehydrated in NF osmotic solution (WL = 0.491 g g^−1^ fresh material and WL = 0.549 g g^−1^ fresh material for OS 1.2 and NF, respectively). Samples treated in OS < 0.45 μm after initial intensive WL start gradually reaching equilibrium. It is consistent with the previous study on pomegranate arils, where it was concluded that OD induces the biggest mass exchange during the first 30 to 60 min of the process [6].

**Figure 4:**
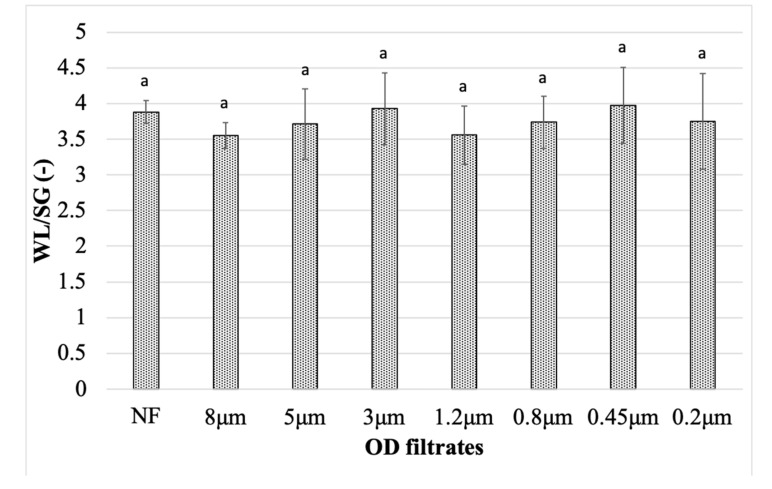
The mean ratio of water loss to solid gain (WL/SG) during osmotic dehydration of pumpkin slices in non-filtrated and filtrated osmotic solutions. Mean values followed by the same letter were not significantly different (*p* < 0.05) according to the HSD Tukey’s least significance difference test.

Effectiveness of osmotic dehydration is shown as WL/SG ratio (Figure 4). Mean WL was over 3.5 times higher than SG for all of the samples. It is consistent with previous studies concerning OD of apples in concentrated chokeberry juice [29]. The lowest WL/SG ratio was obtained when OS 8 μm (WL/SG = 3.55) was applied and the highest ratio was obtained in the case of OS 0.45 μm (WL/SG = 3.97). WL/SG ratio was similar in most cases and there was no significant difference when NF solution was applied. It is inconsistent with previous studies on pumpkin, where WL/SG ratio was higher when high molecular weight solution was used [30]. No significant difference between the results means that overall, filtration had no influence on the mass transfer during the process. However, it should be noted that when shorter time of OD is considered, it is better to use filtrated juices as it leads to the highest WL as well as significantly higher SG.

Modified Penetration model and Peleg’s models were used in the study to describe SG and WL of pumpkin cylinders dehydrated in non-filtrated and filtrated osmotic solutions (Table 2). The good fit of the models was determined on the basis of high values of R^2^ (R^2^ > 0.96 in every case) and low values of RMSE (RMSE < 0.038). Preliminary studies showed that the best model to describe SG is the modified Penetration model. Addition of parameter *B* that describes the shape of the curve to the classic Penetration model resulted in much better fit of SG kinetics when concentrated juice was used as an osmotic solution. Also, this model has been previously successfully used to describe SG in the studies on apples dehydrated in concentrated chokeberry juice [29].

In the case of WL, the best fit demonstrated Peleg’s model which uses two constants, *k*_1_ that illustrates initial mass transfer and *k*_2_ that describes equilibrium moisture content of the sample. As has been previously described by Cichowska et al., low values of *k*_1_ and *k*_2_ indicate both high mass transfer and high water-removal rates [10]. However, there are no clear relationships between the constants in this study. Peleg’s model has been previously used to describe WL in the studies on apples in polyols solutions [31], sugar alcohols and dihydroxyacetone [32], as well as in the case of kiwiberry fruits [33], cherry tomatoes [34] and pumpkin [30].

### 2.3. Properties of Pumpkin Cylinders after Osmotic Dehydration

Table 3 shows total polyphenolics content (TPC) and antioxidant capacity measured by TEAC ABTS and FRAP of pumpkin slices dehydrated in filtrated and non-filtrated chokeberry juice. The initial value of TPC in the fresh sample was 151.66 ± 19.35 mg gallic acid (GA) 100 g^−1^ dm, which is similar to the results obtained in the studies by Lech et al. [18]. OD had a significant influence on the TPC of all the samples. After only 15 min of OD, the TPC increased over 3 times in the case of OS 8 μm to up to almost 4 times in the case of OS 3 μm. This significant increase is due to the high TPC of concentrated chokeberry juice that enhances its values of the pumpkin cylinders. It is consistent with previous research that reported an over 5-fold increase of TPC when chokeberry juice was applied [14]. In the studies on beetroot slices pretreated in concentrated chokeberry juice, it was observed that OD significantly improved the composition of bioactive compounds present in the material [17]. Also, the high rise of TPC is due to the intensive mass transfer at the beginning of the process when influx of osmotic solution had been initiated. Substantial gain of TPC can be seen in each case, regardless of the osmotic solution used.

The application of NF solution resulted in a steady increase of TPC throughout the process. It might be due to the fact that bigger particles have higher total phenolic content, and, with one of the smallest SG, resulted in the highest increase of polyphenolic compounds. In the case of OS 1.2 μm, gain of TPC is moderate and proportional to the SG throughout the process. Decline in TPC content after 90 min in the case of OS 5 μm might be due to the corresponding drop of SG at this time. However, when OS 8 μm was applied, a decrease of TPC was observed that did not resemble high SG at that time. It can be caused by the still broad range of particles in this solution that promote an influx of smaller particles with lower TPC load. Another reason for reduction of polyphenolic compounds might be leaching as a result of hydrolysis of polymers that leads to formation of lower molecular weight compounds [35]. However, in the case of some of the solutions (OS 8 μm and OS 0.2 μm), after an initial increase of TPC and 90 min of the process, a substantial decline can be seen, followed by a subsequent increase of TPC at the end of OD. It might be due to the plasmolysis and resulting uncontrolled influx of osmotic solution. After 120 min, the highest increase of TPC was obtained in the case of NF and OS 1.2 μm.

Antioxidant capacity of the samples was measured using TEAC ABTS and the FRAP method. The results obtained for TEAC ABTS were roughly confirmed by the FRAP method (R^2^ > 0.97 for all variants). However, there was no correlation between TEAC ABTS and TPC. It might be due to the presence of compounds other than polyphenols, i.e., vitamins and volatile compounds, that exhibit the ability to scavenge free radicals and therefore influence the antioxidant capacity values.

A significant increase of TEAC ABTS was noticed in all variants after 15 min of the process—from 3 to almost 4 times higher than at the beginning of the OD. Similar to the initial significant increase of TPC, antioxidant capacity also increased due to the properties of concentrated chokeberry juice that exhibits the ability to scavenge free radicals [20]. It is consistent with research by Lech et al., where chokeberry juice concentrate was used in OD of pumpkin [12]. In most cases, an increase of TEAC ABTS can be noticed from the beginning followed by a decrease in TEAC ABTS values after 60 min of the process for most variants. It might be due to the leaching of bioactive particles as well as selective penetration occurring during OD. Significantly higher TEAC ABTS values obtained in the case of NF solution at the beginning of the process might be due to the presence of high molecular weight compounds, i.e., tannins, that are more potent than simple phenolics [20]. Also, high molecular weight compounds present in NF solution are slower to move and might accumulate at the surface of the sample, hindering the further inflow of particles [27]. At the end of the process, the highest values of TEAC ABTS were obtained in the case of OS 0.45 μm and OS 0.8 μm. It is probably the result of low molecular weight of the compounds present in the osmotic solution and corresponding high osmotic pressure that facilitates plasmolysis, and therefore the influx of antioxidants. Also, smaller particles are faster to move and might penetrate the sample more easily, and as a result, distribute evenly throughout the sample [9].

### 2.4. Properties of Osmotic Solution (OS) after Osmotic Dehydration

Total polyphenolic content and antioxidant capacity measured by two different in vitro tests (TEAC ABTS and FRAP) of osmotic solutions are shown in Table 4. A steady decrease of TPC in NF solution can be seen which is consistent with an increase of TPC in the sample when NF solution was used. In the case of OS 8 μm, a slight increase (by 3%) of TPC can be seen after 15 min followed by a substantial increase (by 16%, *p* < 0.05) after 30 min of the process. These data correlate with the lowest increase of TPC in the pumpkin cylinders in the case of this OS. Overall, decrease of TPC, TEAC ABTS and FRAP, apart from the loss of bioactive compounds penetrating into the osmotically dehydrated material, might be due to the disintegration of native compounds, remaining in osmotic solution, as a result of the long processing time and high temperature [17]. As for an increase of TPC and both TEAC ABTS and FRAP after 60 min of the process, it might be due to the outflow of substances from the material along with high WL occurring at the same time.

Oszmiański and Wojdyło reported that the average TPC of chokeberry juice is 3729.07 mg GA·100 g^−1^ dm [20], which is only slightly higher than the TPC of NF solution before the OD obtained in this study. The heterogenous structure of concentrated chokeberry juice used as osmotic solution as well as the natural differences in biological materials might be the cause of this difference.

High values of TPC and antioxidant capacity in the osmotic solution at the beginning of the process might be due to the selective penetration occurring in the material. From the start, low molecular weight particles take part in the mass transfer as the openings in the plasmodesmata are relatively small, and therefore, bigger particles with higher TPC load remain in the solution, increasing the total phenolic content [14]. It can be seen in the case of OS 8 μm and OS 5 μm at 30 and 60 min of the process, respectively. Then, after plasmolysis, when a wider range of particles flow into the sample, the antioxidant capacity and TPC of OS decrease.

Comparison of TPC at the beginning and after 120 min revealed that in the case of NF, OS 5 μm and OS 0.2 μm, a significant reduction of TPC was obtained. It might be connected with an increase of TPC in the material but also, it might be due to the reduction of temperature-sensitive compounds such as anthocyanins which was also observed in the studies by Nowicka et al. [36]. In other cases, a substantial increase (i.e., by 18% in the case of OS 8 μm, 7% in the case of OS 3 μm and 6.9% in the case of OS 0.45 μm) of TPC after the process was reported. It might be due to the leaching of polyphenols as a result of hydrolysis of polymers that were more prone to flowing out of the sample together with the flow of water [35].

As for antioxidant capacity of osmotic solution measured by FRAP and TEAC ABTS, no correlation has been found between these methods. A similar observation was noted in the case of TPC. It might be due to the differences in these assays as the FRAP method measures the direct capacity of different compounds able to reduce ferric iron [37], whereas ABTS is based on the ability of the compounds to reduce stable ABTS radical cations [38]. The study by Grzesik et al. stated that the main decisive factor of reactivity in the FRAP assay is the presence of the second hydroxyl group in the phenolic ring [39], which might be one of the reasons of discrepancies in the results obtained by TEAC ABTS and FRAP.

## 3. Materials and Methods

### 3.1. Materials

Pumpkin cv. Butternut used in the study was obtained at a local farm (Wroclaw, Poland). Pumpkin was washed and cut into cylinders with a diameter of 18 ± 0.1 mm and 3.35 ± 0.15 mm of thickness. The initial values of moisture content (M_C_), total phenolic content (TPC) and antioxidant activity (measured by ABTS and FRAP) obtained for fresh pumpkin amounted to 90.28%, 151.66 ± 19.35 mg GA·100 g^−1^ dm, 0.31 ± 0.01 mmol Trolox·100 g^−1^ dm and 0.51 ± 0.01 mmol Trolox·100 g^−1^ dm, respectively. Osmotic solution was prepared from commercial concentrated chokeberry juice (Rauch Polska, Płońsk, Poland) according to the procedure described below.

### 3.2. Osmotic Solution

Osmotic solution was filtrated prior to the osmotic dehydration process. Chokeberry juice (65 °Brix) was diluted to 20 °Brix to facilitate the filtration and put through a series of filters, starting from the biggest to the smallest ones. The pore size of the Cellulose Nitrate (CN) Membrane Filters (Sartorius AG, Goettingen, Germany) used in the study were: 8, 5, 3, 1.2, 0.8, 0.45 and 0.2 μm. After each filtration, chemical analysis was conducted and filtrated solution was concentrated to 40 °Brix by evaporation under reduced pressure using a Buchi ROTAVAPOR (BÜCHI Labortechnik AG, Flawil, Switzerland) R-151 (temperature 45 °C, pressure 100 Pa).

### 3.3. Osmotic Dehydration

Osmotic dehydration was performed in a water bath (PS-40A, CE FCC RoHS, China) at 45 ± 2 °C for 2 h. Measurements were made in triplicate and the samples were weighted before the process and after 15, 30, 60, 90 and 120 min. The experiment was carried out using 90 mL of filtered or non-filtrated osmotic solution and 30 g of material. The 3:1 ratio was maintained throughout the process. The samples were drained with tissue paper before each weighting to remove the external moisture. The osmotic dehydration procedure was developed taking into account the results of previous studies [29].

Calculation of water loss (WL), solid gain (SG) and weight reduction (WR) was performed using equations described by Lech et al. [14].

Preliminary studies were performed to choose the best models to describe experimental data. Eventually, the modified Penetration model (Equation (1)) and Peleg’s model (Equation (2)) were used in the study.

(1) $$\mathrm{WL}\;or\;\mathrm{SG}=k\cdot t^{B}$$

The modified Penetration model was previously used in the study by Masztalerz et al. [29], where *k* is diffusion coefficient and parameter *B* describes the shape of the model.

(2) $${\mathrm{WL}}_{}\;or{\;\mathrm{SG}}_{}=\frac{t}{k_{1}+k_{2}\cdot t}$$

Peleg’s model uses parameter *k*_1_ as an initial rate of WL or SG, while parameter *k*_2_ describes the equilibrium value of solid gain or water loss [40].

### 3.4. Physical and Chemical Analysis

#### 3.4.1. Moisture Content (M_C_)

Moisture content of the pumpkin samples was determined by drying at 70 °C for 24 h under reduced pressure using a vacuum dryer (SPT-200; ZEAMiL Horyzont, Krakow, Poland). All measurements were performed in triplicate.

#### 3.4.2. Water Activity (a_w_)

Water activity of the osmotic solutions was measured after filtration. The solution samples were put into AquaLab DewPoint 4TE (Decagon Devices Inc., Pullman, WA, USA) and the measurement was performed at 25 ± 0.5 °C (*n* = 3).

#### 3.4.3. Concentration of Osmotic Solution

The Atago Digital Brix Refractometer PAL-3 (Atago Co., Ltd., Tokyo, Japan) was used to measure the concentration of osmotic solution before and after OD in three repetitions.

#### 3.4.4. Density of Osmotic Solution

The density *ρ* (kg·m^−3^) of osmotic solutions was calculated using Equation (3):

(3) $$\rho =\frac{m}{V}$$

where *m* is the mass measured using analytical balance and *V* is the total volume of the solution determined using HumiPyc 2 Gas Pycnometer (InstruQuest Inc., Coconut Creek, FL, USA). All measurements were performed in triplicate.

#### 3.4.5. Viscosity of Osmotic Solution

Viscosity of osmotic solutions was measured at 45 °C using a Vibro Viscometer SV-10 (A&D Company, Limited, Tokyo, Japan). The measurement was performed before and after OD in 3 repetitions.

#### 3.4.6. Preparation of the Samples for Chemical Analysis

Prior to chemical analysis, all pumpkin samples were dried using a vacuum dryer (SPT-200; ZEAMiL Horyzont, Krakow, Poland) at 40 °C under reduced pressure (100 Pa) for 24 h in order to reach equilibrium moisture content, M_C_ = 5.72%. The extracts were obtained by the sonication (10 min) of 500 mg of samples in 2 mL of 80% aqueous methanol. After being kept for 24 h at 4 °C in the dark, the extracts were once again sonicated (10 min) and centrifuged (1500× *g*; 10 min; 4 °C). The supernatant was submitted to the determination of total polyphenolic contents and antioxidant capacity.

#### 3.4.7. Determination of Total Phenolic Content and Antioxidant Capacity (TEAC ABTS and FRAP Methods)

The Folin–Ciocalteu method was used to determine total polyphenolic content (TPC) of both pumpkin samples and osmotic solution, with gallic acid (GA) used as a standard according to the procedure described by Gao et al. [41]. The antioxidant capacity of extracts and chokeberry juice concentrate used as an osmotic solution was determined by the Trolox Equivalent Antioxidant Capacity test (TEAC ABTS) according to Re et al. [38]. The Ferric Reducing Antioxidant Potential (FRAP) method was conducted in the above-mentioned samples according to Benzie and Strain [37]. All measurements were done in triplicate (*n* = 3) and results were presented as an average of mmol Trolox 100 g dm^−1^ (± standard deviation).

### 3.5. SEM Imaging

Scanning electron microscope (SEM) EVO LS15 (Zeiss, Jena, Germany) was used to characterize the sample captured at the high-magnification images. Dried samples were sprayed with the thin layer of gold and placed into the chamber from which the air has been removed. Sample images were recorded at 300× magnification.

### 3.6. Statistical Analysis

STATISTICA v. 12.0 (StatSoft, Inc., Tulsa, CA, USA) was used to perform statistical analysis. Homogenous groups were established by HSD Tukey’s least significance difference test at a significance level of α = 0.05. Also, one-way analysis of variance (ANOVA) was carried out to analyze the data. Mathematical models were fitted using TableCurve 2D v5.01 (Systat Software, San Jose, CA, USA) on the basis of determination coefficient, R^2^, and root mean square error (RMSE).

## 4. Conclusions

Filtration of osmotic solution (OS) moderately affected mass transfer during osmotic dehydration (OD), however, it resulted in even distribution of the OS inside the sample when solution with smaller particles was applied. The highest solid gain (SG) after 120 min of OD was obtained for the solution filtrated with the membrane of 0.8 μm pore size, whereas when 60 min of the process was considered, the best results were obtained for OS 8 μm. As for the water loss (WL), at the end of the process, the highest WL was obtained for non-filtrated solution, but when shorter time (60 min) of the process was considered, better results were obtained for the 0.2 μm solution.

Application of concentrated chokeberry juice resulted in a significant increase of both total phenolic content and antioxidant capacity measured by FRAP and TEAC ABTS in the sample in each variant. However, significant differences between the variants with NF solution and with filtrates were reported. The highest increase of TPC was obtained in the case of NF solution and OS 1.2 μm, whereas the highest antioxidant capacity (TEAC ABTS) was obtained in the case of OS 0.45 μm and OS 0.8 μm. Also, filtration affected chemical properties of the osmotic solutions during and after the process.

Overall, filtration might not be recommended when 120 min of the process is considered. However, a shorter time of the process (60 min) results in much better quality of dehydrated material, determined as total polyphenolic content (TPC) and antioxidant capacity (measured by FRAP and TEAC ABTS), as well as solid gain and water loss, than when non-filtrated solution is applied.

To sum up, filtration can be a useful tool in osmotic dehydration modelling when heterogenous concentrated juice is used as an osmotic solution.

## Figures and Tables

**Table 1:** Physical and chemical properties of fresh and filtered osmotic solutions before osmotic dehydration.

Osmotic Solution	Water Activity a_w_ (-)	Density kg·m^−3^	Viscosity mPa·s	TPC mg GA·100 g^−1^ dm	TEAC ABTS mmol Trx·100 g^−1^ dm	FRAP mmol Trx·g^−1^ dm
NF	0.9424 ± 0.0008 ^b^	1200.42 ± 13.2 ^a^	2.85 ± 0.08 ^c^	3501.3 ± 102.9 ^b^	49.7 ± 1.22 ^f^	32.9 ± 0.81 ^b^
8 μm	0.9438 ± 0.0001 ^a,b,c^	1189.16 ± 17.84 ^a^	2.77 ± 0.08 ^b,c^	3257.4 ± 56.7 ^a^	38.09 ± 0.94 ^a^	32.9 ± 0.81 ^b^
5 μm	0.9444 ± 0.0005 ^a,c^	1181.25 ± 9.45 ^a^	2.74 ± 0.07 ^a,b,c^	3282.9 ± 73.4 ^a^	38.89 ± 0.96 ^a,b^	32.51 ± 0.8 ^a,b^
3 μm	0.9446 ± 0.0001 ^a,c^	1190.24 ± 20.23 ^a^	2.59 ± 0.07 ^a,b^	3293.4 ± 9.2 ^a^	44.99 ± 1.11 ^d^	30.73 ± 0.76 ^a,b^
1.2 μm	0.9428 ± 0.0008 ^a,b^	1191.53 ± 14.3 ^a^	2.61 ± 0.08 ^a,b^	3361.9 ± 87.5 ^a,b^	33.99 ± 0.84 ^e^	31 ± 0.76 ^a,b^
0.8 μm	0.9434 ± 0.0006 ^a,b^	1187.24 ± 15.43 ^a^	2.62 ± 0.05 ^a,b^	2681.2 ± 8 ^c^	43.29 ± 1.06 ^c,d^	35.26 ± 0.87 ^c^
0.45 μm	0.9429 ± 0.0002 ^a,b^	1188.76 ± 13.08 ^a^	2.61 ± 0.08 ^a,b^	2929.8 ± 8.8 ^d^	39.09 ± 0.96 ^a,b^	30.54 ± 0.75 ^a^
0.2 μm	0.9455 ± 0.0001 ^c^	1194.87 ± 10.75 ^a^	2.56 ± 0.05 ^a^	3374.7 ± 79.5 ^a,b^	40.99 ± 1.01 ^b,c^	30.5 ± 0.75 ^a^

Mean values followed by the same letter were not significantly different (*p* < 0.05) according to the HSD (honest significant difference) Tukey’s least significance difference test; GAE—gallic acid equivalent; TEAC ABTS—Trolox Equivalent Antioxidant Capacity; FRAP—Ferric Reducing Antioxidant Potential; Trx—Trolox.

**Table 2:** Mathematical models with drying constants and coefficient of determination, R^2^, as well as RMSE for SG and WL of osmotically dehydrated samples.

**SG**					
**Model Name**	**Variant**	**Constants**		**R^2^**	**RMSE**
		***k***	***B***		
Modified Penetration model	NF	0.0354	0.2899	0.9968	0.0033
	8 μm	0.0541	0.2014	0.9961	0.0037
	5 μm	0.0249	0.3802	0.9946	0.0046
	3 μm	0.0211	0.4133	0.9934	0.0051
	1.2 μm	0.0398	0.2721	0.9778	0.0089
	0.8 μm	0.0273	0.3556	0.9899	0.0061
	0.45 μm	0.0266	0.3505	0.9773	0.0088
	0.2 μm	0.0373	0.2793	0.9717	0.0098
**WL**					
**Model Name**	**Variant**	**Constants**		**R^2^**	**RMSE**
		***k*_1_**	***k*_2_**		
Peleg’s model	NF	29.563	1.6359	0.9957	0.0149
	8 μm	23.808	1.7922	0.9974	0.0109
	5 μm	24.051	1.8447	0.9974	0.0106
	3 μm	26.845	1.7530	0.9832	0.0135
	1.2 μm	27.262	1.7902	0.9854	0.0253
	0.8 μm	27.231	1.7689	0.9997	0.0034
	0.45 μm	20.354	1.8995	0.9990	0.0066
	0.2 μm	24.260	1.7968	0.9685	0.0384

SG—solid gain; WL—water loss.

**Table 3:** Total polyphenolics content (TPC) and antioxidant capacity measured by TEAC ABTS and FRAP of pumpkin slices dehydrated in filtrated and non-filtrated chokeberry juice.

	Time, min	NF	8 μm	5 μm	3 μm	1.2 μm	0.8 μm	0.45 μm	0.2 μm
TPC mg GA·100 g−1 dm	0	151.66 ± 19.35	151.66 ± 19.35	151.66 ± 19.35	151.66 ± 19.35	151.66 ± 19.35	151.66 ± 19.35	151.66 ± 19.35	151.66 ± 19.35
	15	495.88 ± 18.04 ^a,b^	493.69 ± 9.87 ^a^	560.16 ± 8.99 ^a–e^	604.69 ± 44.21 ^a–h^	510.39 ± 21.26 ^a–c^	566.85 ± 20.95 ^a–f^	496.72 ± 4.64 ^a,b^	544.82 ± 30.38 ^a–d^
	30	834.32 ± 33.26 ^f–k^	626.81 ± 6.75 ^a–h^	654.18 ± 5.93 ^a–h^	677.87 ± 39.65 ^a–h^	603.32 ± 57.9 ^a–h^	609.88 ± 8.74 ^a–h^	571.33 ± 25.8 ^a–g^	631.02 ± 61.97 ^a–h^
	60	861.6 ± 32.71 ^h–k^	799.33 ± 18.03 ^d–k^	771.03 ± 2.57 ^c–k^	691.88 ± 33.02 ^a–h^	697.99 ± 28.23 ^a–i^	767.33 ± 14.99 ^b–j^	803.05 ± 22.9 ^d–k^	789.38 ± 44.33 ^d–k^
	90	969.73 ± 46.24 ^i–k^	738.04 ± 57.29 ^a–i^	711.69 ± 33.76 ^a–i^	808.89 ± 15.48 ^d–k^	837.14 ± 24.77 ^f–k^	864.32 ± 35.48 ^h–k^	782.51 ± 27.09 ^c–k^	731.17 ± 5.37 ^a–i^
	120	1040.32 ± 31.95 ^k^	814.81 ± 35.49 ^d–k^	821.46 ± 13.08 ^e–k^	842.32 ± 358.73 ^g–k^	1017.11 ± 15.45 ^j,k^	788.99 ± 27.4 ^d–k^	805.23 ± 25.62 ^d–k^	822.19 ± 2.81 ^e–k^
TEAC ABTS mmol Trx·100 g−1 dm	0	0.31 ± 0.01	0.31 ± 0.01	0.31 ± 0.01	0.31 ± 0.01	0.31 ± 0.01	0.31 ± 0.01	0.31 ± 0.01	0.31 ± 0.01
	15	10.74 ± 0.26 ^w^	3.82 ± 0.09 ^a^	7.27 ± 0.18 ^l,m^	8.14 ± 0.2 ^n–p^	4.93 ± 0.12 ^c–e^	4.76 ± 0.12 ^b–d^	8.35 ± 0.21 ^o–r^	5.53 ± 0.14 ^e–h^
	30	12.69 ± 0.31 ^x^	6.45 ± 0.16 ^j^	5.98 ± 0.15 ^h–j^	10.75 ± 0.26 ^w^	8.6 ± 0.21 ^p–s^	4.32 ± 0.11 ^a,b^	7.77 ± 0.19 ^m–o^	5.62 ± 0.14 ^f–i^
	60	6.06 ± 0.15 ^h–j^	9.01 ± 0.22 ^s–u^	6.21 ± 0.15 ^i,j^	7.36 ± 0.18 ^l,m^	7.67 ± 0.19 ^l–n^	5.31 ± 0.13 ^c–g^	5.1 ± 0.13 ^c–f^	8.86 ± 0.22 ^r–t^
	90	9.35 ± 0.23 ^u–w^	5.67 ± 0.14 ^f–i^	10.63 ± 0.26 ^w^	6 ± 0.15 ^h–j^	8.39 ± 0.21 ^p,r^	6.5 ± 0.16 ^j–k^	7.4 ± 0.18 ^l,m^	4.73 ± 0.12 ^b–c^
	120	8.21 ± 0.2 ^n–p^	7.45 ± 0.18 ^l,m^	4.07 ± 0.1 ^a^	5.82 ± 0.14 ^g–i^	7.09 ± 0.17 ^k,l^	9.47 ± 0.23 ^u^	9.07 ± 0.22 ^s–u^	5.35 ± 0.13 ^d–g^
FRAP mmol Trx·100 g−1 dm	0	0.51 ± 0.01	0.51 ± 0.01	0.51 ± 0.01	0.51 ± 0.01	0.51 ± 0.01	0.51 ± 0.01	0.51 ± 0.01	0.51 ± 0.01
	15	9.36 ± 0.23 ^u^	3.64 ± 0.09 ^a,b^	5.79 ± 0.14 ^j–l^	6.92 ± 0.17 ^n–p^	4.65 ± 0.11 ^d–f^	4.57 ± 0.11 ^d–f^	6.99 ± 0.17 ^p–s^	4.83 ± 0.12 ^e–h^
	30	9.46 ± 0.23 ^u^	5.05 ± 0.12 ^f–h^	5.93 ± 0.15 ^k,l^	9.4 ± 0.23 ^u^	6.44 ± 0.16 ^m,n^	3.58 ± 0.09 ^a,b^	6.61 ± 0.16 ^o–r^	5.17 ± 0.13 ^g–i^
	60	5.57 ± 0.14 ^i–k^	7.1 ± 0.17 ^o–r^	4.81 ± 0.12 ^d–g^	6.46 ± 0.16 ^g–i^	6.26 ± 0.15 ^l,m^	4.01 ± 0.1 ^b,c^	4.88 ± 0.12 ^e–h^	7.38 ± 0.18 ^p–s^
	90	7.72 ± 0.19 ^o–r^	4.92 ± 0.12 ^e–h^	8.55 ± 0.21 ^t^	5.19 ± 0.13 ^g–i^	6.94 ± 0.17 ^n–p^	4.94 ± 0.12 ^e–h^	5.84 ± 0.14 ^k,l^	4.31 ± 0.11 ^c,d^
	120	7.05 ± 0.17 ^o–r^	6.14 ± 0.15 ^l,m^	3.5 ± 0.09 ^a^	5.32 ± 0.13 ^h–j^	6.26 ± 0.15 ^l,m^	7.36 ± 0.18 ^p–s^	7.47 ± 0.18 ^r,s^	4.53 ± 0.11 ^d,e^

Mean values followed by the same letter in rows and columns within the same method were not significantly different (*p* < 0.05) according to the HSD Tukey’s least significance difference test, excluding the time 0 min. GAE—gallic acid equivalent; TEAC ABTS—Trolox Equivalent Antioxidant Capacity; FRAP—Ferric Reducing Antioxidant Potential; Trx—Trolox.

**Table 4:** Total polyphenolic content (TPC) and antioxidant capacity measured by TEAC ABTS and FRAP of filtrated and non-filtrated osmotic solutions.

	Time, min	NF	8 μm	5 μm	3 μm	1.2 μm	0.8 μm	0.45 μm	**0.2 μm**
TPC mg GA·100 g−1 dm	0	3501.34 ± 102.93 ^k–p^	3257.38 ± 56.66 ^h–m^	3282.94 ± 73.44 ^i–m^	3293.4 ± 9.22 ^i–m^	3361.94 ± 87.5 ^i–o^	2681.18 ± 8.05 ^a–c^	2929.79 ± 8.77 ^c–g^	3374.71 ± 79.5 ^i–o^
	15	3382.66 ± 82.37 ^i–o^	3356 ± 94.78 ^i–o^	3462.63 ± 51.77 ^j–o^	3132.31 ± 18 ^f–i^	3239.77 ± 116.4 ^h–l^	3520.35 ± 91.62 ^l–p^	2638.16 ± 43.22 ^a,b^	3515.33 ± 82.81 ^l–p^
	30	3273.42 ± 125.52 ^i–m^	3996.71 ± 69.46 ^s^	3440.35 ± 23.28 ^j–o^	3388.16 ± 29.11 ^i–o^	3476.6 ± 142.48 ^j–p^	3299.45 ± 118.55 ^i–m^	3634.83 ± 164.66 ^o–r^	3755.5 ± 66.41 ^p–s^
	60	2983.91 ± 27.39 ^d–h^	3252.74 ± 78.61 ^h–m^	3987.43 ± 28.28 ^s^	3142.78 ± 57.67 ^f–i^	2844.76 ± 8.54 ^a–e^	3893.54 ± 61.35 ^r,s^	3269.11 ± 26.8 ^h–m^	2559.59 ± 44.66 ^a^
	90	2739.99 ± 92.88 ^a–d^	3508.94 ± 27.26 ^k–p^	2892.58 ± 114.98 ^b–f^	3136.57 ± 124.74 ^f–i^	3586.06 ± 93.33 ^n–p^	3193.23 ± 9.56 ^g–j^	3343.37 ± 258.64 ^i–m^	3227.48 ± 50.96 ^h–k^
	120	2776.94 ± 85.51 ^a–d^	3983.12 ± 105.8 ^s^	3126.81 ± 53.74 ^e–i^	3541.29 ± 9.91 ^m–p^	3361.37 ± 120.77 ^i–o^	2659.61 ± 43.57 ^a–c^	3146.19 ± 37.14 ^f–i^	2600.88 ± 69.18 ^a^
TEAC ABTS mmol Trx·100 g−1 dm	0	49.7 ± 1.22 ^w^	38.09 ± 0.94 ^g–m^	38.89 ± 0.96 ^i–p^	44.99 ± 1.11 ^t,u^	33.99 ± 0.84 ^c–e^	43.29 ± 1.06 ^s–t^	39.09 ± 0.96 ^i–r^	40.99 ± 1.01 ^l–s^
	15	37.47 ± 0.92 ^f–j^	37.92 ± 0.93 ^f–l^	39.89 ± 0.98 ^j–r^	37.48 ± 0.92 ^f–k^	36.06 ± 0.89 ^c–i^	37.27 ± 0.92 ^f–j^	37.08 ± 0.91 ^e–j^	42.07 ± 1.03 ^p–t^
	30	38 ± 0.93 ^f–m^	48.08 ± 1.18 ^u,w^	41.17 ± 1.01 ^m–s^	41.38 ± 1.02 ^n–s^	41.55 ± 1.02 ^o–s^	32.93 ± 0.81 ^b,c^	35.96 ± 0.88 ^c–i^	30.58 ± 0.75 ^a,b^
	60	35.17 ± 0.86 ^c–g^	38.47 ± 0.95 ^h–o^	47.24 ± 1.16 ^u,w^	37.72 ± 0.54 ^f–k^	36.17 ± 0.89 ^d–i^	39.99 ± 0.98 ^j–r^	35.65 ± 0.88 ^c–h^	38.32 ± 0.94 ^g–n^
	90	32.98 ± 0.81 ^b–d^	43.56 ± 1.07 ^s,t^	37.17 ± 0.91 ^e–j^	38.48 ± 0.95 ^h–o^	37.53 ± 0.92 ^f–j^	37.7 ± 0.93 ^f–k^	34.82 ± 0.86 ^c–f^	29.66 ± 0.73 ^a^
	120	40.73 ± 1 ^k–s^	47.04 ± 1.16 ^u,w^	39.56 ± 0.97 ^j–r^	35.36 ± 0.87 ^c–h^	37.74 ± 0.93 ^f–k^	33.37 ± 0.82 ^b–d^	41.54 ± 1.02 ^o–s^	42.24 ± 1.04 ^r–t^
FRAP mmol Trx·100 g−1 dm	0	32.9 ± 0.81 ^p–s^	32.9 ± 0.81 ^p–s^	32.51 ± 0.8 ^o–s^	30.73 ± 0.76 ^m–p^	31 ± 0.76 ^m–r^	35.26 ± 0.87 ^t,u^	30.54 ± 0.75 ^i–o^	30.5 ± 0.75 ^l–o^
	15	28.04 ± 0.69 ^h–k^	28.76 ± 0.71 ^j–m^	30.36 ± 0.75 ^k–o^	26.34 ± 0.65 ^f–i^	29.92 ± 0.74 ^k–n^	29.63 ± 0.73 ^j–n^	28.05 ± 0.69 ^h–k^	30.68 ± 0.75 ^m–p^
	30	24.27 ± 0.6 ^b–f^	24.66 ± 0.61 ^b–f^	32.95 ± 0.81 ^p–t^	33.45 ± 0.82 ^s–t^	32.98 ± 0.81 ^p–t^	21.75 ± 0.53 ^a^	24.64 ± 0.61 ^b–f^	22.92 ± 0.56 ^a,b^
	60	22.77 ± 0.56 ^a,b^	23.58 ± 0.58 ^a–d^	36.03 ± 0.89 ^u^	27.44 ± 0.43 ^g–j^	25.95 ± 0.64 ^e–h^	30.96 ± 0.76 ^m–r^	28.32 ± 0.7 ^i–l^	29.29 ± 0.72 ^j–n^
	90	23.2 ± 0.57 ^a–c^	23.64 ± 0.58 ^a–e^	23.7 ± 0.58 ^a–e^	29.47 ± 0.72 ^j–n^	26.26 ± 0.65 ^f–i^	29.82 ± 0.73 ^k–n^	25.6 ± 0.63 ^d–g^	21.87 ± 0.54 ^a^
	120	23.18 ± 0.57 ^a–c^	23.62 ± 0.58 ^a–e^	29.29 ± 0.72 ^j–n^	26.3 ± 0.65 ^f–i^	33.08 ± 0.81 ^r–t^	25.3 ± 0.62 ^c–g^	29.12 ± 0.72 ^j–m^	31.62 ± 0.78 ^n–s^

Mean values followed by the same letter in rows and columns within the same method were not significantly different (*p* < 0.05) according to the HSD Tukey’s least significance difference test. GAE—gallic acid equivalent; TEAC ABTS—Trolox Equivalent Antioxidant Capacity; FRAP—Ferric Reducing Antioxidant Potential; Trx—Trolox.
